# Effects of Gamma Irradiation and Supercritical Carbon Dioxide Sterilization on Methacrylated Gelatin/Hyaluronan Hydrogels

**DOI:** 10.3390/jfb14060317

**Published:** 2023-06-08

**Authors:** Christiane Heinemann, Frauke Buchner, Poh Soo Lee, Anne Bernhardt, Benjamin Kruppke, Hans-Peter Wiesmann, Vera Hintze

**Affiliations:** 1 Institute of Materials Science, Max Bergmann Center of Biomaterials, Technische Universität Dresden, Budapester Str. 27, D-01069 Dresden, Germany; 2 Centre for Translational Bone, Joint and Soft Tissue Research, University Hospital Carl Gustav Carus, Faculty of Medicine, Technische Universität Dresden, D-01307 Dresden, Germany

**Keywords:** hydrogels, methacrylated gelatin, methacrylated hyaluronan, human bone marrow stromal cells, osteogenic differentiation

## Abstract

Biopolymer hydrogels have become an important group of biomaterials in experimental and clinical use. However, unlike metallic or mineral materials, they are quite sensitive to sterilization. The aim of this study was to compare the effects of gamma irradiation and supercritical carbon dioxide (scCO_2_) treatment on the physicochemical properties of different hyaluronan (HA)- and/or gelatin (GEL)-based hydrogels and the cellular response of human bone marrow-derived mesenchymal stem cells (hBMSC). Hydrogels were photo-polymerized from methacrylated HA, methacrylated GEL, or a mixture of GEL/HA. The composition and sterilization methods altered the dissolution behavior of the biopolymeric hydrogels. There were no significant differences in methacrylated GEL release but increased methacrylated HA degradation of gamma-irradiated samples. Pore size/form remained unchanged, while gamma irradiation decreased the elastic modulus from about 29 kPa to 19 kPa compared to aseptic samples. HBMSC proliferated and increased alkaline phosphatase activity (ALP) particularly in aseptic and gamma-irradiated methacrylated GEL/HA hydrogels alike, while scCO_2_ treatment had a negative effect on both proliferation and osteogenic differentiation. Thus, gamma-irradiated methacrylated GEL/HA hydrogels are a promising base for multi-component bone substitute materials.

## 1. Introduction

Critical-size bone fractures and poor bone healing due to osteoporosis or bone defects impair mobility and quality of life in an aging, multi-morbid population and put a strain on the healthcare system. These conditions generally require external intervention with bone transplants to augment bone repair and regeneration. However, these are limited, e.g., by their restricted availability, the necessity of two surgical procedures, and high costs [1].

Biopolymeric hydrogels have gained considerable attention in different medical applications, due to their tunable physicochemical properties and their physiological resemblance to the organic part of the extracellular matrix (ECM) providing a hydrophilic three-dimensional environment conductive to cell functions [2]. Further, when supplemented with additional functional compounds like ceramic nano- and microparticles or when used as delivery systems, e.g., for growth factors and antimicrobials, they are particularly effective in promoting tissue regeneration and wound healing [3,4,5,6]. Thus, also in bone tissue engineering, these hydrogels are considered as a highly promising alternative to metal implants and cement [1,2,3,7].

Hydrogels employed in bone regeneration and tissue engineering can be of natural or synthetic origin [2]. The natural polymers collagen type I, its processed form gelatin (GEL), and hyaluronan (HA) have been widely employed due to their good biocompatibility, low immunogenicity and cytotoxicity, as well as their resorbability after metabolic and enzyme-controlled degradation [2,8,9,10].

Hydrogels of GEL methacrylate, as an inexpensive, photocrosslinkable material, displayed osteogenic properties on bone marrow and adipose-derived mesenchymal stem cells (MSC) in vitro and in vivo [11,12,13]. Methacrylated HA-based hydrogels have also been demonstrated to promote the osteogenic differentiation of adipose-derived MSC or osteoblastic cell lines when combined with polycaprolactone nanofibers [14] or arginine-based unsaturated poly (ester amide) [15]. Thus, fabricating hybrid hydrogels is a promising approach for improving material and biological properties [8,16]. In particular, those composed of HA and collagen or GEL are considered as highly relevant biomimetic substrates resembling the organic ECM. Hybrid hydrogels thereof are thought to combine the most favorable characteristics of both biopolymers, which are, e.g., supporting cell adhesion for collagen/GEL and being non-immunogenic and anti-inflammatory for HA [17,18]. Hence, Zhang et al. found superior osteogenic properties of methacrylated collagen/HA hydrogels compared to pure methacrylated HA gels regarding BMSC [18]. Methacrylated GEL/HA hybrid hydrogels with different composition have been studied with respect to their physicochemical properties and the cellular response of endothelial cells [16,17]. Hybrid GEL/HA turned out to be stiffer and contain slightly larger pores compared to pure methacrylated GEL ones, while methacrylated HA conveyed increased swelling and resistance to collagenase digestion [17]. Likewise, Camci-Unal et al. found that the addition of methacrylated GEL into HA improved mechanical properties and increased cell spreading [16]. However, a comparative study on the effects of pure methacrylated HA, methacrylated GEL, and GEL/HA on the proliferation and osteogenic differentiation of hBMSC has not been done so far.

For their clinical application and approval from the regulatory authorities as a bone replacement material, FDA-approved terminal sterilization will be a prerequisite [19]. For biomaterial implants, these include gamma irradiation, electron-beam irradiation, ethylene oxide (EtO) gas, dry heat, and steam heat sterilization [20]. However, biopolymer-containing materials are quite sensitive to standard sterilization procedures like gamma irradiation or heat [19]. Terminal sterilization methods might convey negative or positive effects on material properties like mechanical integrity and biocompatibility [21,22]. Therefore, it is suggested that each system requires case-by-case analysis to select the most suitable technique that leaves the main properties unaltered. Further, in light of the rather limited number of studies in this aspect, further research is needed for a more comprehensive overview on the impact of sterilization with respect to the intrinsic properties of hydrogel systems [19].

It is well known that gamma irradiation causes depolymerization and the formation of large amounts of the carbonyl group in biopolymers like HA and other natural polysaccharides as well as in gelatin via free radicals [23,24,25,26,27,28]. Hydrogels are especially challenging to sterilize due to the presence of water in the hydrogel structure, which can promote physical and chemical alterations of the material depending on the sterilization technique [19].

A promising alternative sterilization method is the application of supercritical CO_2_ (scCO_2_) [20]. At relatively low pressure and temperature, CO_2_ transitions to a supercritical state, combining the characteristics of fluids and gases. Especially noteworthy are the high penetration ability and the dissolving power. Furthermore, scCO_2_ is non-toxic and non-reactive and can easily be removed by depressurization. Therefore, in addition to other applications like extraction and drying, scCO_2_ treatment has been proposed for the inactivation of microorganisms [29] and is a promising approach for sterilizing sensitive biomedical materials [30]. Low amounts of volatile low-molecular-weight organic additives can be included in the process to increase the inactivation rate of microorganisms. ScCO_2_ has been successfully applied for the sterilization of different biomaterials, including allografts, synthetic, and natural-based scaffolds, as well as technical textiles [31]. In most cases, the structural and mechanical properties of the materials were not changed after the treatment. The compressive strength of alginate hydrogels sterilized by scCO_2_, for example, was shown to be significantly higher compared to that of gamma-irradiated alginate hydrogels [22]. With respect to methacrylated gelatin hydrogels, a recent study by Zhang et al. evaluated the effects of autoclaving and ethylene oxide (EtO) gas as well as ethanol treatment, commonly used in a laboratory setting, on hydrogel properties. These were morphology, swelling behavior, and elastic modulus, as well as macrophage gene expression in vitro [32]. While the physicochemical properties were only marginally altered, the sterilization technique had a significant impact on gene expression. Rizwan et al. investigated the effect of three FDA-approved terminal sterilization methods (autoclaving, EtO gas, and gamma irradiation) on mechanical properties and biodegradation as well as cellular response towards methacrylated GEL hydrogels submerged in PBS during sterilization [21]. Gamma irradiation increased the stiffness, reduced the pore size, and significantly reduced the degradation rate, while autoclaving and EtO gas reduced the stiffness but did not modify degradation. Here, only EtO treatment significantly decreased fibroblast viability, while cell adhesion and spreading were unaltered for all investigated sterilization techniques. Thus, the choice of the terminal sterilization technique can strongly influence physicochemical and biological properties of methacrylated GEL and hydrogels in general. To the best of our knowledge, there has been no study so far comparing the impact of gamma irradiation and scCO_2_-treatment on methacrylated (HA)- and GEL-based hydrogels, as well as hybrids thereof regarding their physicochemical properties and the biological response with respect to human bone marrow-derived mesenchymal stem cells (hBMSC).

In the present study, we investigated the impact of gamma irradiation and scCO_2_-treatment on the abovementioned properties for three different photo-crosslinked hydrogel variants. These are composed of either methacrylated HA, methacrylated GEL, or GEL/HA hybrids thereof. The aim was to assess differences in enhancing bone cell functions between the materials and the possible influence of the particular sterilization techniques thereof. In this context, the hydrolytic degradation of the hydrogels, as well as their mechanical and surface properties, were determined. Further, the proliferation and osteogenic differentiation of hBMSC were assessed. Thus, the novelty of this study is that it compares for the first time the effects of the named gel combinations and different terminal sterilization methods on the physicochemical properties and the biological response of hBMSC. Thus, it contributes important knowledge to our still rather limited understanding of sterilization effects on hydrogel properties. The outcome of this study was expected to clarify which gel variant is the most suitable in promoting bone cell functions of hBMSC and which sterilization techniques leave this hydrogel property mainly unaltered. This should identify the most promising gel variant and sterilization technique as a base for combinatory approaches with other osteogenic compounds and their prospective application as bone replacement material in vivo.

## 2. Materials and Methods

### 2.1. Materials

**Figure 1:**
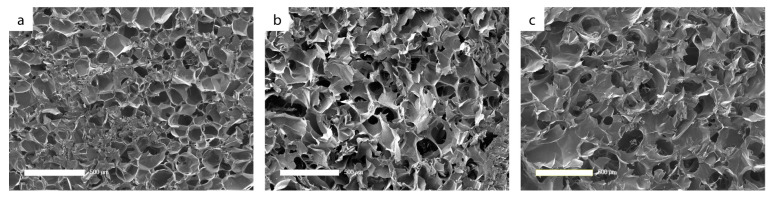
SEM images of cross-sections of the freeze-dried hydrogels based on methacrylated GEL (**a**), GEL/HA (**b**), and HA (**c**) in aseptic condition. Bar: 500 µm.

**Figure 2:**
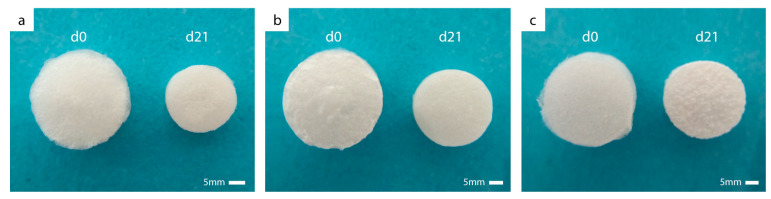
Photographs of lyophilized hydrogels in aseptic condition based on methacrylated GEL (**a**), GEL/HA (**b**), and HA (**c**) at the initial state (d0) and after 21 days of incubation in PBS (d21).

**Figure 3:**
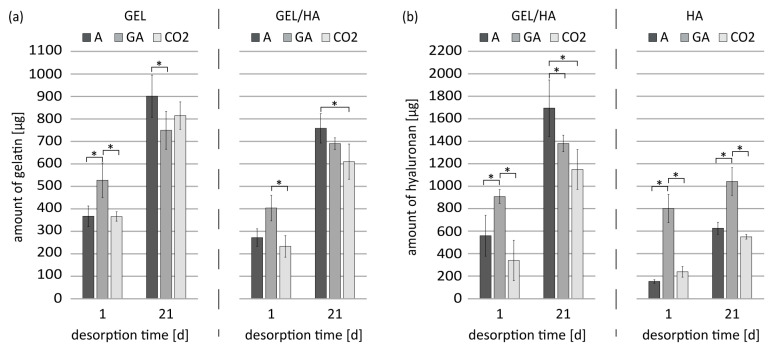
Hydrolytic degradation of methacrylated GEL, GEL/HA, and HA hydrogels in aseptic (A), gamma-irradiated (GA), and scCO_2_-sterilized (CO_2_) conditions. Degradation was conducted in PBS. The release of GEL (**a**) and HA (**b**) was determined. Data represent mean values with standard deviation (n = 3). Statistics: Two- and three-way analysis of variance (ANOVA); * indicate *p* < 0.05 was considered statistically significant.

**Figure 4:**
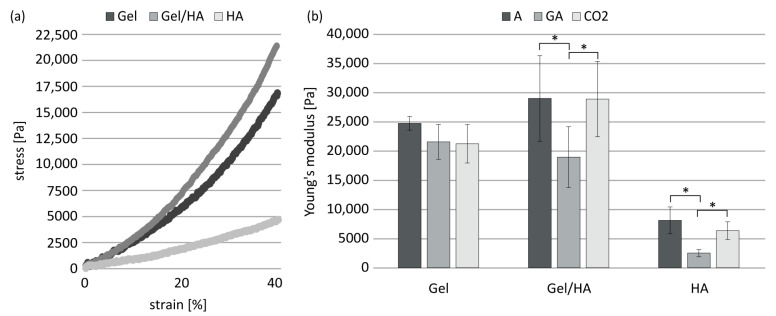
Stress–strain curves of hydrogels based on methacrylated GEL, GEL/HA, and HA in aseptic condition (**a**). Young’s modulus of GEL, GEL/HA, and HA hydrogels in aseptic (A), gamma-irradiated (GA), and scCO_2_-treated (CO_2_) conditions as determined by compressive loading (**b**). Data represent mean values with standard deviation (n = 4). Statistics: Two- and three-way analysis of variance (ANOVA); * indicate *p* < 0.05 was considered statistically significant.

**Figure 5:**
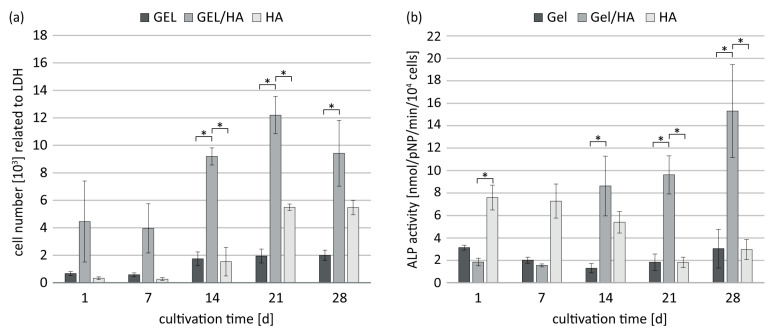
Cell number (**a**) and specific ALP activity (**b**) of osteogenically induced hBMSC cultivated on methacrylated GEL, GEL/HA, and HA hydrogels prepared under aseptic conditions. Cell number was assessed by LDH activity. Data represent mean values with standard deviation (n = 3). Statistics: Two- and three-way analysis of variance (ANOVA); * indicate *p* < 0.05 was considered statistically significant.

**Figure 6:**
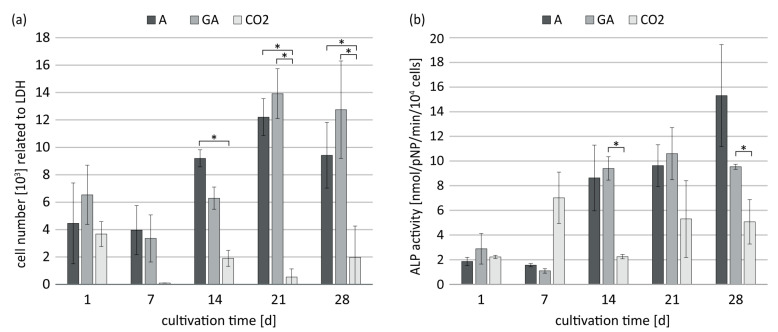
Cell number (**a**) and specific ALP activity (**b**) of osteogenically induced hBMSC cultivated on methacrylated GEL/HA hydrogels aseptic (A), gamma-irradiated (GA), and scCO_2_-treated (CO_2_) conditions. Cell number was assessed by LDH activity. Data represent mean values with standard deviation (n = 3). Statistics: Two- and three-way analysis of variance (ANOVA); * indicate *p* < 0.05 was considered statistically significant.

Methacrylated GEL (product no. 5208) was purchased from Advanced BioMatrix (Carlsbad, CA, USA). Lithium phenyl-2,4,6-trimethyl-benzoylphosphinate (LAP, >98%, product no. L0290) was obtained from TCI Deutschland GmbH (Eschborn, Germany). Methacrylated HA was kindly provided by Dr. Stephanie Möller and Dr. Matthias Schnabelrauch (Innovent e.V., Jena, Germany) and prepared from native HA (cosmetic grade, 99.2%, MW = 1400 kDa, product no. 5110010900) from Streptococcus, Kraeber & Co. GmbH, Ellerbek, Germany) as previously described [33]. For keeping sample designation in figures simple, methacrylated HA is indicated as HA and methacrylated GEL is indicated as GEL in Figure 1, Figure 2, Figure 3, Figure 4, Figure 5 and Figure 6 as well as Figure A3 and Figure A4.

### 2.2. Preparation of Hydrogels

In this study, three different types of hydrogels were prepared aseptically: 5% methacrylated GEL, 3% methacrylated HA, and 5% GEL/3% HA (GEL/HA). Firstly, 5% GEL, 3% HA, and 5% GEL/3% HA were dissolved in deionized water. In order to enable photo-crosslinking, 10 mg/mL of the photoinitiator, LAP, was added in a ratio of 1:10 (*v*/*v*) and thoroughly mixed with the above-mentioned solutions [34]. Cylinder-shaped scaffolds with a diameter of 5 mm were prepared by pipetting 75 µL of each solution into silicone molds. After crosslinking by UV irradiation (365 nm, 0.17 W/cm^2^, 10 min), the samples were frozen at −80 °C for 30 min and then freeze-dried in a Martin Christ Epsilon 2–4 LSC device. Afterward, they were rinsed twice in deionized water over a period of 60 min, during which time they were swollen and freeze-dried a second time.

### 2.3. Sterilization of Hydrogels

Freeze-dried hydrogels were either sterilized by supercritical CO_2_ or gamma irradiation with 30 kGy according to DIN EN ISO 11137-1 (BBF Sterilisationservice GmbH, Kernen, Germany). Before scCO_2_ treatment, the samples were sealed into Tyvek^®^/foil bags. ScCO_2_ treatment was performed in a pre-cooled (4 °C) 320 mL stainless steel autoclave (Carl Roth) equipped with an inlet and outlet valve, manometer, and safety valve. The sealed samples were inserted into the autoclave and 0.2 mL of acetic anhydride, as well as 0.2 mL of hydrogen peroxide (37%) (both from Sigma-Aldrich, Burlington, MA, USA), were pipetted to the bottom of the autoclave. Subsequently, the autoclave was filled with liquid CO_2_ (200 g). The liquid CO_2_ was transferred to the supercritical state by heating the autoclave to 38 °C at 8.5 MPa pressure. These conditions were held for 30 min, followed by depressurization. In order to verify the transition of CO_2_ into the supercritical phase, an indicator was treated along with the samples in each run, as described before [22].

### 2.4. Hydrolytic Degradation Experiments

In order to assess the stability of hydrogels in aqueous solution, the release of hydrogel components was studied after incubation in Dulbecco’s Phosphate Buffered Saline (PBS; Sigma-Aldrich, Taufkirchen, Germany) at 37 °C for up to 21 days. At specific time points, the 500 µL supernatant of each sample was collected for analysis and replaced with fresh PBS of the same volume.

Lowry assay: Gelatin degradation was analyzed using the Lowry method as described before [35]. In brief, 40 µL of supernatant was mixed with 200 µL of 98 vol% 2% (*w*/*v*) sodium carbonate in 0.1 N NaOH and 2 vol% 0.5% (*w*/*v*) copper sulfate in 1% (*w*/*v*) sodium citrate. A reaction time of 15 min was applied in the dark, before 20 µL Folin reagent (Sigma-Aldrich, Taufkirchen, Germany) was added, followed by additional incubation for 80 min in the dark. A calibration curve was prepared from a graded series of a gelatin reference. The absorbance was measured at 700 nm using a microplate reader (infinite M200Pro, Tecan, Switzerland).

Turbidity measurement: The amount of HA in the supernatants was analyzed by turbidimetric measurements using HA/cetyltrimethylammonium bromide (CTAB) complexes [36]. Briefly, 50 µL supernatant was mixed with 50 µL acetate buffer and incubated for 5 min at 37 °C. Subsequently, 100 µL of CTAB reagent (2.5 g CTAB dissolved in 100 mL of 2% (*w*/*v*) NaOH) was added, mixed, and the absorbance was measured immediately at 600 nm wavelength using the Tecan plate reader. A calibration curve was obtained from a graded series of HA.

### 2.5. Mechanical Characterization

The elastic modulus of the hydrogels as determined with a Microsquisher^®^ (Cellscale, Waterloo, ON, Canada). The hydrogels were analyzed after swelling the freeze-dried samples for 24 h in PBS at room temperature. The height of the hydrogels was measured with the integrated digital camera and compressed to 70% of their original height to derive a stress–strain plot. The slope of the stress–strain curve was used to determine the elastic modulus.

### 2.6. Scanning Electron Microscopy

The morphology of the freeze-dried gels was investigated by scanning electron microscopy (SEM, Philips ESEM XL 30, FEI, 3 kV high-vac mode) via secondary electron detector. Slices of freeze-dried hydrogels were mounted on aluminum stubs and sputter coated (MED010, FA. Balzers, Balzers, Liechtenstein) with carbon (Plano, Wetzlar, Germany).

### 2.7. Cell Culture Experiments

HBMSC were kindly provided by Prof. Bornhäuser and coworkers, Medical Clinic I, University Hospital Dresden. The cells were isolated from the bone marrow aspirate of a 25-year-old male donor and expanded. Passage 5 was used for the experiments of the present study. The hBMSC were cultured in alpha medium (α-MEM) supplemented with 10% fetal calf serum (FCS), 100 U/mL penicillin, and 100 µg/mL streptomycin in a humidified atmosphere at 37 °C and 5% CO_2_. The medium and all supplements were purchased from Merck (Berlin, Germany).

In preparation for cell seeding, the hydrogels were placed in 48-well plates and soaked in the cell culture medium for 24 h. After medium replacement, hBMSC were seeded with a density of 20,000 cells per 48 well. Four days after seeding, the cells were osteogenically induced by adding 10 nM dexamethasone (Sigma-Aldrich), 50 µM ascorbic acid-2-phosphate (Sigma-Aldrich), and 5 mM β-glycerophosphate (Sigma-Aldrich) to the medium. In the further course of the experiment, the medium was changed twice weekly.

### 2.8. Colorimetric Measurements

Proliferation and osteogenic differentiation were assessed by lactate dehydrogenase (LDH) and alkaline phosphatase (ALP) assay, respectively. All measurements were performed with cell lysates obtained after 1, 7, 14, 21, and 28 days of cultivation using 1% Triton X-100 (Sigma-Aldrich) in PBS. LDH activity was determined from the total activity of LDH in the cell lysates using the LDH cytotoxicity detection kit (Takara, Saint-Germain-en-Laye, France). An aliquot of the cell lysate was mixed with LDH substrate buffer and the enzymatic reaction was stopped after 30 min with 0.5 M HCl. Likewise, cell lysates of defined cell numbers were used to prepare the calibration curve. For analysis, the absorbance at 492 nm wavelength was measured using the Tecan microplate reader. ALP activity was determined from the total activity of ALP in the cell lysates using p-nitrophenyl phosphate as substrate. For this purpose, an aliquot of the cell lysate was added to the ALP substrate buffer containing 2 mg/mL p-nitrophenyl phosphate (Sigma-Aldrich), 0.1 M diethanolamine, 1 mM MgCl_2_, and 0.1% Triton X-100 (pH 9.8). The mixture was incubated at 37 °C for 30 min before the enzymatic reaction was stopped by adding 0.5 M NaOH. A calibration curve was prepared from different concentrations of p-nitrophenol. Finally, the absorbance was measured at 405 nm with the Tecan microplate reader.

### 2.9. Statistics

All data were recorded in at least triplicates and expressed as mean ± standard deviation. Two- and three-way analyses of variance (ANOVA) with Tukey Post-Hoc test were performed where applicable to assess significant differences. Here, *p*-values < 0.05 are considered significant. Only the statistically significant measurement results were marked as such in the graphs, even if further analyses between, e.g., material classes and time periods were carried out.

## 3. Results

### 3.1. Morphology of Hydrogels

**Figure A1:**
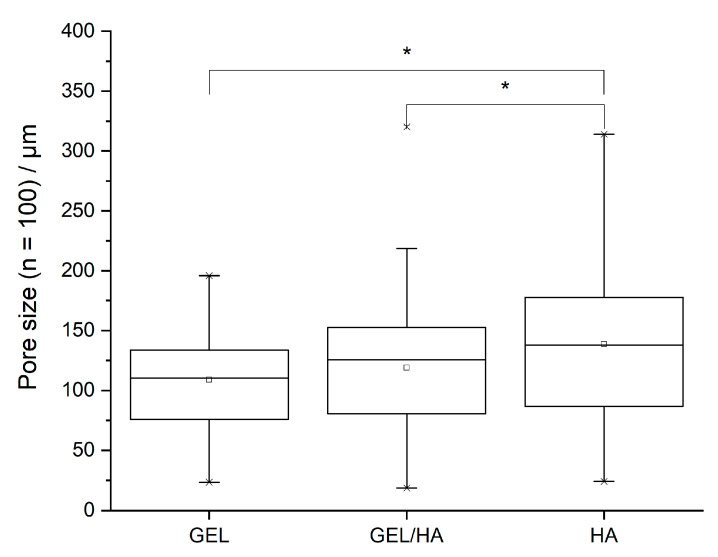
Pore size of hydrogels based on methacrylated GEL, GEL/HA and HA in aseptic conditions. Data represent mean values with standard deviation (n = 3). Statistics: Two- and three-way analysis of variance (ANOVA); * indicate *p* < 0.05 was considered statistically significant.

**Figure A2:**
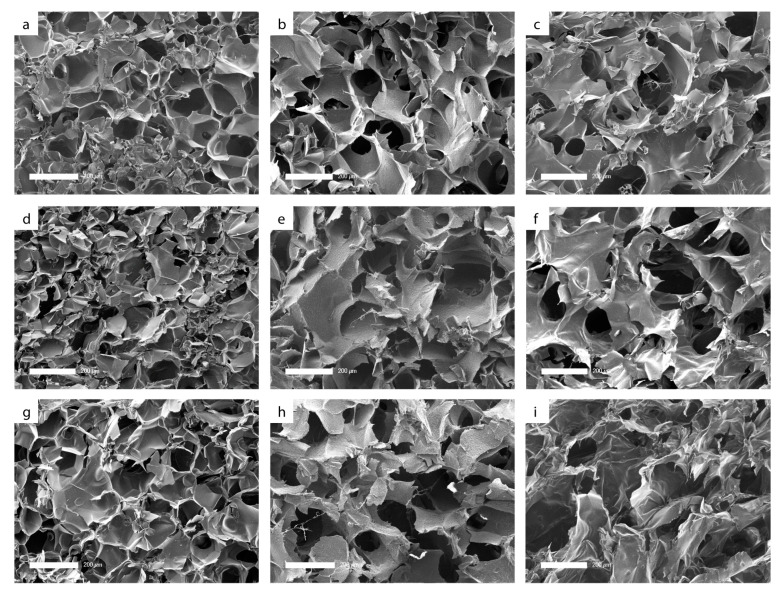
SEM images of cross-sections of the freeze-dried hydrogels based on methacrylated GEL (**a**,**d**,**g**), GEL/HA (**b**,**e**,**h**), and HA (**c**,**f**,**i**) in aseptic (**a**–**c**), gamma-irradiated (**d**–**f**), and scCO_2_-sterilized (**g**–**i**) conditions. Bar: 200 µm.

Investigating the surface morphology by SEM, lyophilized hydrogels in aseptic condition (before sterilization) revealed similar pore shape and distribution for all modifications (Figure 1). The pore size increases with increasing amount of HA in the hydrogel. While the pore sizes for GEL tend to be smaller (about 110 µm), the mean pore size for GEL/HA increases to 120 µm, and for HA to 140 µm (Figure A1). Sterilization by gamma irradiation or scCO_2_ had no effect on the size and shape of the pores (Figure A2).

### 3.2. Hydrolytic Degradation of Hydrogels

After 21 days of incubation in PBS, an apparent volume loss compared to the initial state was visible macroscopically for all hydrogels due to hydrolytic degradation. The aseptically produced hydrogels are exemplarily shown in Figure 2.

The influence of the sterilization methods on the degradation of the hydrogels was determined quantitatively by detecting the degradation products, GEL, and HA (Figure 3).

Considering the gelatin release (Figure 3a), it can be generally stated that GEL samples lost a slightly higher amount of gelatin than the GEL/HA samples, irrespective of the sterilization method. Based on the initial mass of gelatin (3750 µg), about 24% of the gelatin was released from GEL hydrogels in aseptic condition after 21 days in PBS. This is in agreement with the macroscopically visible mass loss of the hydrogels after 21 days (Figure 2). In combination with HA, the release of gelatin was reduced to 20% in GEL/HA. This difference in gelatin release between GEL and GEL/HA samples was already apparent at d1.

Interestingly, for both hydrogel types, gamma irradiation, but not scCO_2_ sterilization, caused a significantly higher initial degradation, which was about 13% for GEL and 11% for GEL/HA after d1. However, after 21 days, the percentage of released gelatin was about 20% for GEL and 18% for GEL/HA. This was, in the case of GEL samples, significantly lower than corresponding GEL samples in aseptic condition. Furthermore, samples sterilized by scCO_2_ also displayed a slightly reduced release of gelatin after 21 days compared to samples in aseptic condition, which was significant in the case of GEL/HA. 

Regarding the release of HA (Figure 3b), the GEL/HA samples released higher amounts than the pure HA hydrogels, irrespective of the sterilization method. After 21 days, approximately 75% of HA incorporated in the hydrogel (2250 µg) was released from hydrogels in aseptic condition. In contrast, only 27% of HA in the HA hydrogels was released. Gamma irradiation, but not scCO_2_ treatment, had a significant effect on the release of HA, with 35% release from HA hydrogels on d1 after incubation in PBS, as compared to 8% for the non-sterilized samples (A). This difference was also apparent at d21 with 45% versus 27% release, indicating the particular sensitivity of pure HA hydrogels to gamma irradiation. A similar but less pronounced effect was demonstrated by the GEL/HA samples on d1 only. Here, gamma irradiation led to a comparable loss of HA for GEL/HA and HA hydrogels. At d21, however, gamma-irradiated GEL/HA displayed a significantly lower release than samples in aseptic condition. In addition, for the scCO_2_-treated hydrogels, the HA release was significantly lower than for aseptic gels at this time point.

### 3.3. Mechanical Properties of Hydrogels

The elastic moduli of the hydrogels were determined by compressive loading (Figure 4a,b). The stress–strain curves of the hydrogels GEL, HA and GEL/HA could be clearly distinguished (Figure 4a). GEL hydrogels and GEL/HA composites displayed a compressive modulus of up to 29 kPa, about 3.5 times higher than the HA hydrogels. In the case of GEL, the hydrogels lose 15% of their elasticity due to both sterilization methods, with more deformation occurring under load. For GEL/HA, a reduction from 29 kPa to about 19 kPa was only observed after gamma irradiation. This was also the case for the HA hydrogels. Here, the elastic modulus decreased by more than half compared to hydrogels in aseptic condition.

### 3.4. Cell Proliferation and Differentiation of hBMSC

When hBMSC was cultivated on the aseptically prepared hydrogels, the amount of metabolically active cells was highest on GEL/HA samples at every time point of cultivation. The differences in cell number compared to GEL and HA samples were significant at the later time points of cultivation (Figure 5a). Further, from day 14 onwards, there was a significant increase in specific ALP activity, indicating osteogenic differentiation in the GEL/HA hydrogels, which increases by a factor of 7 from d1 compared to d28 (Figure 5b). In contrast, cells on pure GEL hydrogels showed no increase in ALP activity. The cells cultivated on HA gels had the highest specific ALP activity on d1 and d7, which declined from d14 onwards. Thus, GEL/HA composites were found to be most favorable in promoting the cellular functions of hBMSC, in terms of proliferation and osteogenic differentiation.

In the following, the influence of gamma irradiation and supercritical scCO_2_ treatment were analyzed with respect to proliferation and ALP activity of hBMSC in GEL/HA composites (Figure 6a,b). Gamma irradiation marginally altered the number of metabolically active cells and ALP activity compared to corresponding aseptically prepared samples. In contrast, the scCO_2_ treatment led to a significant decrease in cell number. Accordingly, ALP activity was significantly diminished on d14 and d28 compared to the aseptic and gamma-irradiated samples. 

**Figure A3:**
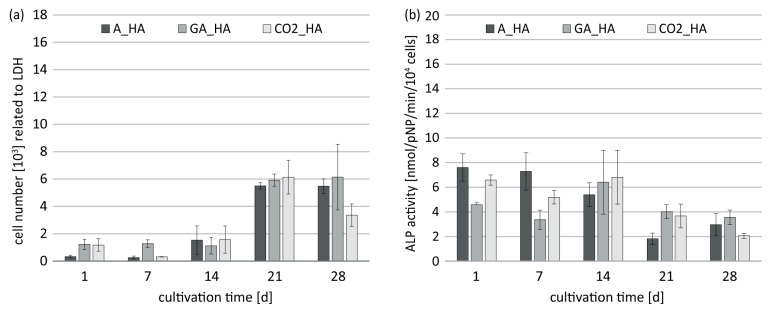
Cell number (**a**) and specific ALP activity (**b**) of osteogenically induced hBMSC cultivated on hydrogels based on methacrylated HA in aseptic (A), gamma-irradiated (GA), and scCO_2_-sterilized (CO_2_) conditions. Cell number was assessed by LDH activity. Data represent mean values with standard deviation (n = 3). Statistics: Two- and three-way analysis of variance (ANOVA).

**Figure A4:**
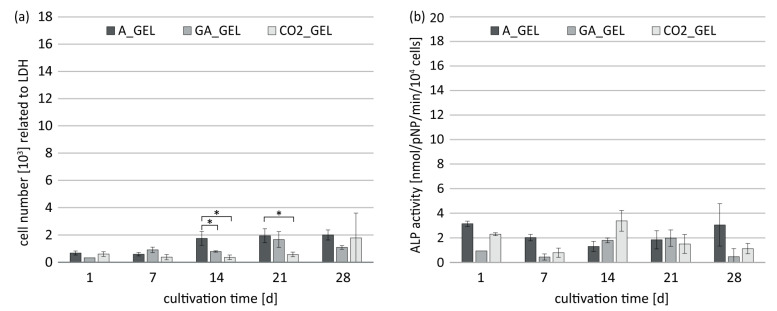
Cell number (**a**) and specific ALP activity (**b**) of osteogenically induced hBMSC cultivated on hydrogels based on methacrylated GEL in aseptic (A), gamma-irradiated (GA), and scCO_2_-sterilized (CO_2_) conditions. Cell number was assessed by LDH activity. Data represent mean values with standard deviation (n = 3). Statistics: Two- and three-way analysis of variance (ANOVA); * indicate *p* < 0.05 was considered statistically significant.

Interestingly, pure HA gels did not show a decreased number of metabolically active cells when sterilized with scCO_2_ (Figure A3). However, the cell number on scCO_2_-sterilized methacrylated GEL hydrogels was significantly lower compared to gamma-irradiated and aseptic hydrogels (Figure A4).

## 4. Discussion

Biopolymeric hydrogels are considered as a promising alternative to present ceramic and metal-based materials in bone tissue engineering, due to their tunable physicochemical properties and their biomimetic features resembling the organic ECM [1]. The biopolymers HA and GEL, the denatured form of collagen, have become particularly attractive in constructing hybrid hydrogels that combine the favorable characteristics of each. The preparation of GEL/HA hybrid hydrogels with different methacrylated HA and methacrylated GEL concentrations—but not exactly the same composition as in the present study—and their impact on the mechanical properties and swelling behavior as well as endothelial cell spreading were recently studied [16,17]. However, a comparative study on the effects of pure methacrylated HA, methacrylated GEL, and the hybrid GEL/HA on the proliferation and osteogenic differentiation of hBMSC has not been undertaken so far.

Further, for clinical applications, sterilization is a mandatory prerequisite, but might have a negative effect on the material properties [37]. Therefore, in this study, the impact of gamma irradiation and scCO_2_ treatment on the physicochemical and biological properties of methacrylated GEL/HA composite hydrogels was investigated in comparison to their respective pure variants.

The pore sizes of the lyophilized gel variant before sterilization were comparable for methacrylated GEL/HA and HA gels, while those of methacrylated GEL tended to be smaller. This is in line with findings of Velasco-Rodriguez demonstrating that low methacrylated HA concentrations in hybrid hydrogels slightly enlarged pore sizes compared to pure methacrylated GEL hydrogels [17]. It was suggested that HA adsorbs more water and thus the ice crystals expanding during freezing yielded larger voids. As expected, there was no effect of sterilization on pore sizes of lyophilized gels.

The hybrid methacrylated GEL/HA hydrogels exhibit, with about 25 kPa for the aseptically treated gel, a higher stiffness than the pure modifications. In the present study, pure HA hydrogels exhibit the lowest stiffness, which is about three times lower than for the other two gel types. Nevertheless, the addition of HA to GEL slightly increased the compressive moduli as previously reported by Camci-Unal et al. [16]. In the latter study, the Young’s modulus of the hybrid gel (5% GEL, 2% HA) was about 30 kPa, similar to our results, while those of the pure gels were below 10 kPa. Furthermore, Velasco-Rodriguez et al. investigated hydrogels with different GEL/HA ratios and suggested that methacrylated GEL/HA hybrids have additional mechanical stability compared to their pure counterparts due to the physical bonds (hydrogen bonding, electrostatic interactions), next to chemical cross-links, between the well-intermixed polymer chains [17]. In this study, the Young’s moduli for hybrids gels with comparable composition were only between 5.6 and 9.0 kPa and below the range of the methacrylated GEL/HA gel investigated in the present study.

For all hydrogel types, gamma irradiation led to a significant reduction in stiffness, in particular for methacrylated HA-containing gels. For methacrylated GEL, this is in contrast to the findings of Rizwan et al., where gamma irradiation led to significant stiffer scaffolds based on methacrylated GEL compared to unsterilized gels, which was attributed to additional crosslinking of gelatin chains due to radiation [21,38]. However, the major difference between our studies is that Rizwan et al. submerged their gels in PBS during gamma irradiation, while our gels were freeze-dried. In the latter case, a reduction in the Young’s modulus of GEL-based samples is caused by the cleavage of polymer chains at a radiation dose of 25 kGy [39]. In HA-containing materials, gamma irradiation might cause chain scission triggered by free radicals, which can lead to depolymerization and reduction in molecular size, which in turn affects mechanical properties [23,24,25,26,40]. The present findings regarding reduced stiffness due to gamma irradiation are in line with the results of the hydrolytic hydrogel degradation. Here, gamma irradiation led to an increased release of the individual components—methacrylated GEL and methacrylated HA—in the supernatant, which was significant at d1 for all hydrogel types and, in case of pure methacrylated HA gels, for both time points (d1 and d21). In contrast, scCO_2_ treatment did not have a significant influence on the mechanical properties, as previously described for other hydrogel systems [22,37], which is in line with its marginal influence on initial hydrogel degradation. 

The cell culture examination of hydrogels in an aseptic state revealed that methacrylated GEL/HA exhibits significantly higher proliferation and osteogenic differentiation compared to pure hydrogel variants. In line with this, Zhang et al. found an improved proliferation and osteogenic differentiation of BMSC on methacrylated collagen/HA hybrid gels compared to pure methacrylated HA ones [41]. Likewise, Jha et al. reported that human MSC, cultured on methacrylated HA hydrogels with gelatin-containing HA particles, formed complex 3D cell structures and differentiated towards pre-osteoblasts without osteogenic supplements. In contrast, human MSC cultured without the addition of gelatin displayed a round morphology and did not show osteogenic differentiation, but rather developed towards adipocytes [42]. These observations are consistent with our results with hBMSC on hydrogels of methacrylated HA exhibiting a low proliferation rate, with cell numbers only increasing on day 21, and decreasing ALP activity. Thus, limitations of the pure HA hydrogels are their non-adhesive nature with respect to cell adhesion and spreading [14,16]. However, this can be compensated by a combination of suitable chemical partners to improve osteogenic properties [9,14,42]. In the present context, it can be suggested that gelatin provides cell binding sites via their RGD sequences, allowing cellular spreading, which is considered as a prerequisite for osteogenic differentiation [14,18]. In line with this, Camci-Unal et al. describe that hydrogels composed of methacrylated HA only did not induce cellular adhesion or spreading of HUVEC [16]. Only the addition of methacrylated GEL improved cell spreading due to its cell adhesive functional groups. Furthermore, the improved cellular response was attributed to the higher stiffness achieved by including methacrylated GEL [16]. Similarly, Wang et al. showed that human MSC on stiffer gelatin-hydroxyphenylpropionic acid hydrogels have a higher proliferation rate, larger spreading area, more organized cytoskeletons, more stable focal adhesions, and faster migration rate [43]. In our case, this is true for GEL/HA, displaying the highest stiffness in the present study. However, when comparing HA vs. GEL, pure GEL exhibits the highest stiffness but lower cell numbers than on the pure HA hydrogels, at least on day 21 and day 28. Therefore, stiffness and cell adhesion sites alone are not the only factors influencing cellular response. Likewise, Celikkin et al. reported declining cell numbers for rat BMSC grown on methacrylated GEL hydrogels with no major difference between a 5% and 10% gels [12]. However, in contrast to our study, it was also found that hydrogels composed of pure methacrylated GEL were a suitable environment for osteogenic differentiation of rat-derived BMSC or adipose-derived stem cells [11,12]. Reasons for deviating results might be the difference in material composition, MSC source, but also higher concentrations of osteogenic supplements used in these studies. It also cannot be excluded that cell proliferation in these gels might be influenced by donor variability, which was neither assessed in the cited studies nor here.

Gamma irradiation of the hydrogels did not affect the respective cellular functions. In contrast, treatment with scCO_2_ led to a decrease in cell number over time. Osteogenic differentiation also decreased significantly. It has been stated that the addition of hydrogen peroxide, polyacrylic acid, acetic anhydrides, and other processing adjuncts can provoke changes in the polymer structure [20]. Meyer et al. likewise observed a decreased cytocompatibility on collagen films and collagen sponges after scCO_2_ sterilization. The latter displayed a higher cytotoxic effect than the foils [44]. Hodder et al. suggested that the chemical additives used in sCO_2_ sterilization provide low cell survival. For this reason, samples should be ventilated prior to contact with cells. However, an optimal duration for this has not yet been determined. Further, significant amounts of hydrogen peroxide could still be detected 7 days after sterilization. Even after 14 days, traces could still be found, while only after 21 days there was no hydrogen peroxide detectable. This illustrates that sterilization via scCO_2_ still leaves significant room for optimization before clinical application for biomaterials [45]. In the present study, the samples were used after a ventilating period of 14 days. Therefore, it is possible that trace amounts of hydrogen peroxide could have caused the poor cell survival rate in the scCO_2_-treated hydrogels. Thus, the outgassing time after scCO_2_-treatment should be optimized separately for each material. 

Another aspect that should not be overlooked is the influence of free radicals on cell viability. Kanjickal et al. demonstrated that a significantly higher concentration of free radicals is produced after gamma irradiation and scCO_2_ treatment in PEG hydrogels than for non-sterilized samples. The concentration of free radicals increases with time for all treatments, except for the gamma-irradiated sample, and the increase was significantly higher for the sample sterilized with scCO_2_ [46]. This might already have a strong impact on biocompatibility, since higher concentrations of free radicals can decrease cell viability by increasing intracellular oxidative stress [47]. In addition, due to the UV irradiation of the photoinitiator during the preparation of the samples, free radicals are generated, which trigger the radical addition reaction between the methacrylate side chains resulting in gelation [48]. Possibly, the free radicals remaining after gelation in combination with scCO_2_ treatment additionally increase this fraction. This leads to an imbalance in antioxidants with free radicals, resulting in oxidative stress, which will affect the long-term cell viability and functionality [49].

Thus, the reasons for the cellular decline on scCO_2_-sterilized hydrogels found in the present study may be complex and require further investigation. However, further studies are worthwhile, since low-temperature scCO_2_ treatment, which is increasingly applied for temperature-sensitive natural polymers in current research, still has many advantages over standard procedures [22,31,50,51]. Nevertheless, based on the present results, this method of sterilization is not recommended for GEL- and HA-based hydrogels. If the reason for the effects is indeed the insufficient outgassing time, it would be first necessary to test again which outgassing time has to be applied before starting a cell experiment again. Thus, an outgassing time of 14 days may not be long enough to ensure sufficient biocompatibility. However, it must be kept in mind that long processing periods are undesirable for clinical use as well. Here the use of antioxidants, like Vitamin E doping, might be an alternative solution to improve outcomes [20]. Regarding cellular responses, we did not find any detrimental effects of gamma irradiation on the GEL- and HA-based hydrogels prepared in this study.

We recognized that the present study has potential limitations, such as investigating only one setting of gamma irradiation and scCO_2_ treatment with no variation in dose or outgassing time. Further, change in mechanical parameters over time due to material disintegration and cellular actions was not considered. Finally, UV irradiation time and LAP concentration as a source of free radicals was not varied in these experiments, nor was free radical formation investigated. All these aspects should thus be considered in future studies to gain a more comprehensive knowledge on the impact of these parameters. In summary, gamma-irradiated methacrylated GEL/HA hybrid hydrogels were found to be most suitable as a base for combinatory approaches with further osteogenic compounds and their prospective application as bone replacement material in vivo.

## 5. Conclusions

This study aimed to compare the effects of composition and sterilization methods on the physicochemical and osteogenic properties of methacrylated GEL- and HA-based hybrid hydrogels as well as their respective pure variants. Two sterilization methods—gamma irradiation and scCO_2_ treatment—were used and compared to aseptically prepared hydrogels. The results showed that gamma-irradiated GEL/HA hydrogels are favorable for multi-component bone substitute materials, with enhanced hBMSC proliferation and osteogenic differentiation. In contrast, scCO_2_ treatment had a negative impact on both proliferation and osteogenic differentiation. These findings highlighted the importance for cautious selection of sterilization methods for biopolymeric hydrogels in medical applications. Further, methacrylated GEL/HA hydrogels provide a promising asset in treating bone defects, in particular when combined with other osteogenic components, such as ceramic particles. The in vivo relevance of these materials should be addressed in future studies utilizing an animal model for impaired bone healing.

## Data Availability

The raw data supporting this study´s findings are available upon reasonable request from the corresponding authors.

## References

[1] Elkhoury K., Morsink M., Sanchez-Gonzalez L., Kahn C., Tamayol A., Arab-Tehrany E. (2021). Biofabrication of Natural Hydrogels for Cardiac, Neural, and Bone Tissue Engineering Applications. Bioact. Mater..

[2] Bai X., Gao M., Syed S., Zhuang J., Xu X., Zhang X.-Q. (2018). Bioactive Hydrogels for Bone Regeneration. Bioact. Mater..

[3] Divyashri G., Badhe R.V., Sadanandan B., Vijayalakshmi V., Kumari M., Ashrit P., Bijukumar D., Mathew M.T., Shetty K., Raghu A. (2022). V Applications of Hydrogel-based Delivery Systems in Wound Care and Treatment: An Up-to-date Review. Polym. Adv. Technol..

[4] Utech S., Boccaccini A.R. (2016). A Review of Hydrogel-Based Composites for Biomedical Applications: Enhancement of Hydrogel Properties by Addition of Rigid Inorganic Fillers. J. Mater. Sci..

[5] Kanungo S., Gupta N., Rawat R., Jain B., Solanki A., Panday A., Das P., Ganguly S. (2023). Doped Carbon Quantum Dots Reinforced Hydrogels for Sustained Delivery of Molecular Cargo. J. Funct. Biomater..

[6] Zheng J., Zhao F., Zhang W., Mo Y., Zeng L., Li X., Chen X. (2018). Sequentially-Crosslinked Biomimetic Bioactive Glass/Gelatin Methacryloyl Composites Hydrogels for Bone Regeneration. Mater. Sci. Eng. C.

[7] Xue X., Hu Y., Deng Y., Su J. (2021). Recent Advances in Design of Functional Biocompatible Hydrogels for Bone Tissue Engineering. Adv. Funct. Mater..

[8] Xu Q., Torres J.E., Hakim M., Babiak P.M., Pal P., Battistoni C.M., Nguyen M., Panitch A., Solorio L., Liu J.C. (2021). Collagen-and Hyaluronic Acid-Based Hydrogels and Their Biomedical Applications. Mater. Sci. Eng. R Rep..

[9] Zhai P., Peng X., Li B., Liu Y., Sun H., Li X. (2020). The Application of Hyaluronic Acid in Bone Regeneration. Int. J. Biol. Macromol..

[10] Dong Z., Yuan Q., Huang K., Xu W., Liu G., Gu Z. (2019). Gelatin Methacryloyl (GelMA)-Based Biomaterials for Bone Regeneration. RSC Adv..

[11] Fang X., Xie J., Zhong L., Li J., Rong D., Li X., Ouyang J. (2016). Biomimetic Gelatin Methacrylamide Hydrogel Scaffolds for Bone Tissue Engineering. J. Mater. Chem. B.

[12] Celikkin N., Mastrogiacomo S., Jaroszewicz J., Walboomers X.F., Swieszkowski W. (2018). Gelatin Methacrylate Scaffold for Bone Tissue Engineering: The Influence of Polymer Concentration. J. Biomed. Mater. Res. Part A.

[13] Piao Y., You H., Xu T., Bei H.-P., Piwko I.Z., Kwan Y.Y., Zhao X. (2021). Biomedical Applications of Gelatin Methacryloyl Hydrogels. Eng. Regen..

[14] Patel M., Koh W.-G. (2020). Composite Hydrogel of Methacrylated Hyaluronic Acid and Fragmented Polycaprolactone Nanofiber for Osteogenic Differentiation of Adipose-Derived Stem Cells. Pharmaceutics.

[15] Zhou Y., Gu Z., Liu J., Huang K., Liu G., Wu J. (2020). Arginine Based Poly (Ester Amide)/Hyaluronic Acid Hybrid Hydrogels for Bone Tissue Engineering. Carbohydr. Polym..

[16] Camci-Unal G., Cuttica D., Annabi N., Demarchi D., Khademhosseini A. (2013). Synthesis and Characterization of Hybrid Hyaluronic Acid-Gelatin Hydrogels. Biomacromolecules.

[17] Velasco-Rodriguez B., Diaz-Vidal T., Rosales-Rivera L.C., García-González C.A., Alvarez-Lorenzo C., Al-Modlej A., Domínguez-Arca V., Prieto G., Barbosa S., Soltero Martinez J.F.A. (2021). Hybrid Methacrylated Gelatin and Hyaluronic Acid Hydrogel Scaffolds. Preparation and Systematic Characterization for Prospective Tissue Engineering Applications. Int. J. Mol. Sci..

[18] Zhang T., Chen H., Zhang Y., Zan Y., Ni T., Liu M., Pei R. (2019). Photo-Crosslinkable, Bone Marrow-Derived Mesenchymal Stem Cells-Encapsulating Hydrogel Based on Collagen for Osteogenic Differentiation. Colloids Surf. B Biointerfaces.

[19] Galante R., Pinto T.J.A., Colaco R., Serro A.P. (2018). Sterilization of Hydrogels for Biomedical Applications: A Review. J. Biomed. Mater. Res. Part B Appl. Biomater..

[20] Herczeg C.K., Song J. (2022). Sterilization of Polymeric Implants: Challenges and Opportunities. ACS Appl. Bio Mater..

[21] Rizwan M., Chan S.W., Comeau P.A., Willett T.L., Yim E.K.F. (2020). Effect of Sterilization Treatment on Mechanical Properties, Biodegradation, Bioactivity and Printability of GelMA Hydrogels. Biomed. Mater..

[22] Bernhardt A., Wehrl M., Paul B., Hochmuth T., Schumacher M., Schütz K., Gelinsky M. (2015). Improved Sterilization of Sensitive Biomaterials with Supercritical Carbon Dioxide at Low Temperature. PLoS ONE.

[23] Ahmad A.F., Mohd H.M.K., bin Ayob M.T.M., Rosli N.R.A.M., Mohamed F., Radiman S., Rahman I.A. (2014). Effect of Gamma Irradiation on Hyaluronic Acid and Dipalmitoylphosphatidylcholine (DPPC) Interaction. Proceedings of the AIP Conference Proceedings.

[24] Huang Y.-C., Huang K.-Y., Lew W.-Z., Fan K.-H., Chang W.-J., Huang H.-M. (2019). Gamma-Irradiation-Prepared Low Molecular Weight Hyaluronic Acid Promotes Skin Wound Healing. Polymers (Basel).

[25] Lin C.-Y., Kuo P.-J., Lin Y.-H., Lin C.-Y., Lin J.C., Chiu H.-C., Hung T.-F., Lin H.-Y., Huang H.-M. (2022). Fabrication of Low-Molecular-Weight Hyaluronic Acid&ndash;Carboxymethyl Cellulose Hybrid to Promote Bone Growth in Guided Bone Regeneration Surgery: An Animal Study. Polymers (Basel).

[26] Kuo P.-J., Yen H.-J., Lin C.-Y., Lai H.-Y., Chen C.-H., Wang S.-H., Chang W.-J., Lee S.-Y., Huang H.-M. (2021). Estimation of the Effect of Accelerating New Bone Formation of High and Low Molecular Weight Hyaluronic Acid Hybrid: An Animal Study. Polymers (Basel).

[27] Burton B., Gaspar A., Josey D., Tupy J., Grynpas M.D., Willett T.L. (2014). Bone Embrittlement and Collagen Modifications Due to High-Dose Gamma-Irradiation Sterilization. Bone.

[28] Stanca M., Gaidau C., Zaharescu T., Balan G.-A., Matei I., Precupas A., Leonties A.R., Ionita G. (2023). Physico-Chemical Changes Induced by Gamma Irradiation on Some Structural Protein Extracts. Biomolecules.

[29] Spilimbergo S., Bertucco A. (2003). Non-thermal Bacterial Inactivation with Dense CO_2_. Biotechnol. Bioeng..

[30] White A., Burns D., Christensen T.W. (2006). Effective Terminal Sterilization Using Supercritical Carbon Dioxide. J. Biotechnol..

[31] Ribeiro N., Soares G.C., Santos-Rosales V., Concheiro A., Alvarez-Lorenzo C., García-González C.A., Oliveira A.L. (2020). A New Era for Sterilization Based on Supercritical CO_2_ Technology. J. Biomed. Mater. Res. Part B Appl. Biomater..

[32] Zhang F., Scull G., Gluck J.M., Brown A.C., King M.W. (2022). Effects of Sterilization Methods on Gelatin Methacryloyl Hydrogel Properties and Macrophage Gene Expression in Vitro. Biomed. Mater..

[33] Becher J., Moeller S., Schnabelrauch M. (2017). Building Blocks for Artificial Extracellular Matrices Based on Cross-Linkable Polysaccharide and Glycosaminglycan Sulfates. Proceedings of the Materials Science Forum.

[34] Rother S., Galiazzo V.D., Kilian D., Fiebig K.M., Becher J., Moeller S., Hempel U., Schnabelrauch M., Waltenberger J., Scharnweber D. (2017). Hyaluronan/Collagen Hydrogels with Sulfated Hyaluronan for Improved Repair of Vascularized Tissue Tune the Binding of Proteins and Promote Endothelial Cell Growth. Macromol. Biosci..

[35] Miron A., Rother S., Huebner L., Hempel U., Käppler I., Moeller S., Schnabelrauch M., Scharnweber D., Hintze V. (2014). Sulfated Hyaluronan Influences the Formation of Artificial Extracellular Matrices and the Adhesion of Osteogenic Cells. Macromol. Biosci..

[36] Song J.-M., Im J.-H., Kang J.-H., Kang D.-J. (2009). A Simple Method for Hyaluronic Acid Quantification in Culture Broth. Carbohydr. Polym..

[37] Bento C.S.A., Gaspar M.C., Coimbra P., de Sousa H.C., Braga M.E.M. (2023). A Review of Conventional and Emerging Technologies for Hydrogels Sterilization. Int. J. Pharm..

[38] Cataldo F., Ursini O., Lilla E., Angelini G. (2008). Radiation-Induced Crosslinking of Collagen Gelatin into a Stable Hydrogel. J. Radioanal. Nucl. Chem..

[39] Amadori S., Torricelli P., Rubini K., Fini M., Panzavolta S., Bigi A. (2015). Effect of Sterilization and Crosslinking on Gelatin Films. J. Mater. Sci. Mater. Med..

[40] Sintzel M.B., Merkli A., Tabatabay C., Gurny R. (1997). Influence of Irradiation Sterilization on Polymers Used as Drug Carriers—a Review. Drug Dev. Ind. Pharm..

[41] Xu C., Su P., Wang Y., Chen X., Meng Y., Liu C., Yu X., Yang X., Yu W., Zhang X. (2010). A Novel Biomimetic Composite Scaffold Hybridized with Mesenchymal Stem Cells in Repair of Rat Bone Defects Models. J. Biomed. Mater. Res. A.

[42] Jha A.K., Xu X., Duncan R.L., Jia X. (2011). Controlling the Adhesion and Differentiation of Mesenchymal Stem Cells Using Hyaluronic Acid-Based, Doubly Crosslinked Networks. Biomaterials.

[43] Wang L.-S., Boulaire J., Chan P.P.Y., Chung J.E., Kurisawa M. (2010). The Role of Stiffness of Gelatin–Hydroxyphenylpropionic Acid Hydrogels Formed by Enzyme-Mediated Crosslinking on the Differentiation of Human Mesenchymal Stem Cell. Biomaterials.

[44] Meyer M., Prade I., Leppchen-Fröhlich K., Felix A., Herdegen V., Haseneder R., Repke J.-U. (2015). Sterilisation of Collagen Materials Using Hydrogen Peroxide Doted Supercritical Carbon Dioxide and Its Effects on the Materials Properties. J. Supercrit. Fluids.

[45] Hodder E., Duin S., Kilian D., Ahlfeld T., Seidel J., Nachtigall C., Bush P., Covill D., Gelinsky M., Lode A. (2019). Investigating the Effect of Sterilisation Methods on the Physical Properties and Cytocompatibility of Methyl Cellulose Used in Combination with Alginate for 3D-Bioplotting of Chondrocytes. J. Mater. Sci. Mater. Med..

[46] Kanjickal D., Lopina S., Evancho-Chapman M.M., Schmidt S., Inbaraj J.J., Cardon T.B., Lorigan G.A. (2009). Electron Spin Resonance Studies of the Effects of Sterilization on Poly (Ethylene Glycol) Hydrogels. J. Biomed. Mater. Res. Part A An Off. J. Soc. Biomater. Jpn. Soc. Biomater. Aust. Soc. Biomater. Korean Soc. Biomater..

[47] Lee G.M., Kim S., Kim E.M., Kim E., Lee S., Lee E., Park H.H., Shin H. (2022). Free Radical-Scavenging Composite Gelatin Methacryloyl Hydrogels for Cell Encapsulation. Acta Biomater..

[48] Xiang L., Cui W. (2021). Biomedical Application of Photo-Crosslinked Gelatin Hydrogels. J. Leather Sci. Eng..

[49] Pahoff S., Meinert C., Bas O., Nguyen L., Klein T.J., Hutmacher D.W. (2019). Effect of Gelatin Source and Photoinitiator Type on Chondrocyte Redifferentiation in Gelatin Methacryloyl-Based Tissue-Engineered Cartilage Constructs. J. Mater. Chem. B.

[50] Bento C.S.A., Alarico S., Empadinhas N., de Sousa H.C., Braga M.E.M. (2022). Sequential ScCO_2_ Drying and Sterilisation of Alginate-Gelatine Aerogels for Biomedical Applications. J. Supercrit. Fluids.

[51] Soares G.C., Learmonth D.A., Vallejo M.C., Davila S.P., González P., Sousa R.A., Oliveira A.L. (2019). Supercritical CO_2_ Technology: The next Standard Sterilization Technique?. Mater. Sci. Eng. C.

